# Resource-oriented interventions for patients with severe mental illnesses in low- and middle-income countries: trials in Bosnia-Herzegovina, Colombia and Uganda

**DOI:** 10.1186/s12888-019-2148-x

**Published:** 2019-06-14

**Authors:** Stefan Priebe, Catherine Fung, Sana Z. Sajun, Racheal Alinaitwe, Domenico Giacco, Carlos Gómez-Restrepo, Alma Džubur Kulenoviĉ, Noeline Nakasujja, Sandra Milena Ramírez, Sabina Slatina, Nelson K. Sewankambo, Hana Sikira, Miguel Uribe, Victoria Jane Bird

**Affiliations:** 1 Unit for Social and Community Psychiatry (WHO Collaborating Centre for Mental Health Services Development), Queen Mary University of London, Newham Centre for Mental Health, E13 8SP, London, UK; 2 Department of Psychiatry, Makerere University College of Health Sciences, Kampala, Uganda; 3 Department of Clinical Epidemiology and Biostatistics and Psychiatry and Mental Health, Pontifica Universidad Javeriana, Hospital Universitario San Ignacio, Bogotá, Colombia; 4 Clinical Center University of Sarajevo, Sarajevo, Bosnia and Herzegovina; 5 Department of Clinical Medicine, Pontificia Universidad Javeriana, Cali, Colombia; 6 School of Medicine, Makerere University College of Health Sciences, Kampala, Uganda; 7 Department of Social and Preventive Medicine and Psychiatry and Mental Health, Pontificia Universidad Javeriana, Bogotá, Colombia

**Keywords:** Global mental health, Psychosocial interventions, Resource-oriented approach, LMICs, Solution-focused, Volunteer support, Family involvement, Severe mental illness

## Abstract

**Background:**

Severe mental illness (SMI) presents a major burden to societies worldwide. Low- and middle-income countries (LMICs) often do not have sufficient financial resources and qualified staff to provide extensive specialised services for outpatients with SMI. Our research therefore aims to explore and test low-cost interventions that use existing resources in routine patient-clinician meetings, families and communities.

**Methods:**

In Bosnia-Herzegovina, Colombia and Uganda, three psychosocial interventions will be tested, i.e. making patient-clinician meetings therapeutically effective through DIALOG+, family involvement in multi-family group meetings, and support for patients in befriending schemes with volunteers. All interventions will be provided to patients with SMI, delivered over a six-month period and evaluated with assessments at baseline and after six and 12 months. We will conduct nine trials including non-controlled trials, non-randomised controlled trials and randomised controlled trials (RCTs). Core outcome criteria will be used across all studies. However, details of study delivery and additional outcome criteria vary to accommodate local contexts, interests and priorities. The studies will be analysed separately, but with the option to compare and combine findings.

**Discussion:**

The approach provides the opportunity to learn from commonalities and differences in the results and experiences across the three resource-oriented approaches and the three countries. If successfully implemented the studies can lead to more extensive research and are expected to inform health policies and clinical practice of community care for patients with SMI in the three participating countries and other LMICs.

**Trial registration:**

All RCTs were registered prospectively and non-randomised trials retrospectively within the ISRCTN Registry.

DIALOG+ in Uganda: ISRCTN25146122 (Date of Registration: 20/11/2018, prospective); DIALOG+ in Colombia: ISRCTN83333181 (Date of Registration: 20/11/2018, prospective); DIALOG+ in Bosnia-Herzegovina: ISRCTN13347129 (Date of Registration: 20/11/2018, prospective); Volunteer Support in Uganda: ISRCTN86689958 (Date of Registration: 04/03/2019, retrospective); Volunteer Support in Colombia: ISRCTN72241383 (Date of Registration: 04/03/2019, retrospective);Volunteer Support in Bosnia-Herzegovina: ISRCTN51290984 (Date of Registration: 20/11/2018, prospective); Family Involvement in Uganda: ISRCTN78948497 (Date of Registration: 04/03/2019, retrospective); Family Involvement in Colombia: ISRCTN11440755 (Date of Registration: 04/03/2019, retrospective); Family Involvement in Bosnia-Herzegovina: ISRCTN13347355 (Date of Registration: 20/11/2018, prospective).

## Background

In order to address global challenges, significant amounts of funding have been made available in high-income countries (HICs) in the last five years to conduct research with and in low- and middle-income countries (LMICs). Much of this funding has been used to support research in global health, including global mental health [1]. There is no overall accepted consensus on what exactly global mental health research should entail. One approach is that research should aim to test and implement evidence-based interventions in LMICs, most of which have exclusively been studied in HICs [2]. Such an approach has limitations: a) using current evidence-based interventions in a different context might be inappropriate and ineffective, particularly as most interventions in mental health care have only small to medium effect sizes; b) many interventions, such as the majority of formal psychological treatments, require significant funding and qualified professional staff, none of which is usually available in LMICs; and c) in various LMICs there are likely to be resources in individuals, families and communities, that may not exist to the same extent in HICs but can be utilised to help people alleviate or overcome the distress of mental disorders.

We therefore applied to the National Institute of Health Research (NIHR) in the United Kingdom (UK) to pursue a different approach. We were awarded funding for a NIHR Global Health Research Group on Developing Psycho-Social Intervention for Mental Health Care at Bart’s and the London School of Medicine and Dentistry (hereby referred to as The Group). The Group aims to establish partnerships of experts in HICs and LMICs, work in collaboration with local stakeholders to develop resource-oriented interventions for people with severe mental illness, and to conduct studies to test these interventions [3]. The interventions are intended to be low-cost and mainly use existing resources and social structures in LMICs. Therefore, if found to be effective, the interventions should facilitate sustainable implementation of effective community care for large numbers of people with severe mental illness (SMI). The three existing resources that we will utilise are a) contacts and meetings between patients and clinicians in the community as they occur in the given context; b) families and friends of patients; and c) volunteers in the community who are willing to spend time with and support patients with SMI. The research designs were all adapted and agreed with local stakeholders to ensure they fit with the local context, priorities and mental health needs. Through the work of the Group, we also intend to increase the expertise in research on psychosocial interventions in the partner countries and more generally to advance the conceptual understanding of what global mental health research may mean and achieve.

The Group consists of partners in Sarajevo (Bosnia-Herzegovina), Bogotá (Colombia), Kampala (Uganda) and London (UK). Bosnia-Herzegovina, Colombia and Uganda are all LMICs, all have aims to expand community care for people with SMI and all share a recent history of armed conflicts. Yet, they are on three different continents and have different traditions and cultures. This may facilitate the learning from commonalities and differences in experiences.

## Methods

### Interventions and study designs

The research conducted by the Group will test three aforementioned interventions - making patient-clinician meetings therapeutically effective through DIALOG+, family involvement in multi-family group meetings, and support for patients in befriending schemes with volunteers. Each intervention is tested in each country, i.e. there are nine studies in total. The study designs include non-controlled trials, non-randomised controlled trials and smaller and larger randomised controlled trials (RCTs). The main characteristics of the nine studies are summarised in Table 1, indicating core consistencies and differences in study designs across the three LMICs.

**Table 1:** Main characteristics of the nine studies that will be conducted in Bosnia-Herzegovina, Colombia and Uganda

	Bosnia-Herzegovina	Colombia	Uganda
DIALOG+	Cluster RCT, 72 patients with depression and anxiety, 14 clinicians, Control group = TAU	Cluster RCT, 168 patients with SMI, 14 clinicians, Control group = TAU	Cluster RCT, 168 patients with MNS, 14 clinicians, Active control group
Family Involvement	RCT, 72 patients with SMI, 36–72 family members/friends, 6–12 clinicians, Control group = TAU	Non-controlled trial, 30 patients with SMI, 30–60 family members/friends, 6–12 clinicians, No control group	Controlled trial, 30 patients with SMI, 30–60 family members/friends, 6–12 clinicians, Control site
Volunteer Support	RCT, 72 patients with SMI, 36 Volunteers, Control group = TAU	Non-controlled trial, 30 patients with SMI, 20 volunteers, No control group	Controlled trial, 30 patients with SMI, 10 volunteers, Control Site

*Abbreviations: RCT* Randomised controlled trial, *SMI* Severe mental illness, *MNS* Mental, neurological and substance misuse disorders, *TAU* Treatment as usual

For each intervention (described below) there is some evidence for effectiveness from HICs, although that evidence is not always conclusive and does not necessarily apply to the precise form of the intervention as used in this research. Yet, all interventions can refer to theories for why they might be beneficial to patients and have been found by local stakeholders in each country to be relevant for the given context.

#### DIALOG+

DIALOG+ has been developed to turn routine meetings between a patient and mental health professional into a therapeutically effective intervention. It is based on quality of life research, concepts of patient-centred communication, developments in information technology, and components of solution-focused therapy. Supported by an App using a tablet computer, it delivers assessment, planning, intervention and evaluation in one procedure.

In the meeting with the mental health professional, patients rate their satisfaction with eight quality of life domains, and three treatment domains. The ratings are summarised on-screen, allowing for comparisons with ratings from any previous meeting. Patients can then decide which life and/or treatment domains they would like to discuss in the current meeting. Each selected life domain or treatment domain is addressed in a four-step solution-focused approach. The four steps are: 1) understanding (Why is the patient dissatisfied? What is nevertheless working well?); 2) looking forward (What is the best case scenario? What is the smallest step forward?); 3) exploring options (What can the patient, the mental health professional and others do?); and finally 4) agreeing on actions (What should be done between now and the next meeting?). The agreed actions are briefly documented and revisited at the beginning of the next meeting.

In an RCT in London, DIALOG+ was compared with an active control condition and was shown to lead to better quality of life, lower symptom levels, more favourable objective social situation and reduced health care costs for patients with schizophrenia [4–6].

DIALOG+ will be tested in three cluster randomised controlled trials, in which mental health professionals and their patients, i.e. clusters, are randomly allocated either to the experimental or the control group. This design will prevent potential contamination effects between the groups and ensure that no professional who is trained in DIALOG+ administers it to a patient in the control group. DIALOG+ will be delivered by mental health professionals, once per month over a period of 6 months, as part of usual routine clinical meetings.

In each country, DIALOG+ will be administered to outpatients with SMI, diagnosed using International Classification of Diseases, Tenth Revision (ICD-10) criteria [7] by mental health professionals but with slight variations in the diagnosis and patient numbers based on the priorities expressed by national stakeholders and reflecting differences in national health and social care systems.

In Uganda, 168 patients with SMI defined as having been diagnosed with psychosis, bipolar, and/or mood affective disorders, (ICD-10 F20–29, F30-F39), epilepsy and/or comorbid substance abuse and 14 mental health professionals including nurses, social workers, psychiatric clinical officers, psychologists and psychiatrists will be part of the trial. The RCT in Uganda will comprise of an active control arm. Patients in the control group will also receive an intervention on a tablet, where they will be asked to complete the DIALOG scale which includes only satisfaction ratings, with minimal input from clinicians. This will be done at the end of their routine sessions so that the ratings will not influence the communication during the meeting.

In Colombia, 168 patients with SMI defined as having been diagnosed with psychosis, bipolar, and/or mood affective disorders (ICD-10 F20–29, F31, F32) will be included and all 14 mental health professionals will be qualified psychiatrists or psychiatric residents.

In Bosnia, a RCT will be conducted with 72 patients with severe depression and anxiety (ICD-10 F30-F39, F40-F49, excluding bipolar disorder) and 14 mental health professionals who will be qualified psychiatrists, psychologists or psychiatric residents.

#### Family involvement

Involving family members or friends in patient’s care can help to improve family communication, overall care and outcomes. The intervention will draw from the tradition of trialogue and psychosis-seminars, where mutual learning occurs through the sharing of experiences, support and psychoeducation on pre-agreed topics [8, 9]. In multi-family group meetings, approximately five to six patients with one or two family members or friends each and one or two mental health professionals will meet and discuss subjects that are defined and selected by the participants. The family intervention sessions will be held once per month, over six months at local community or health centres, or easily accessible locations.

The meetings will normally be chaired by a mental health professional, but the group can also decide to have a patient or family member to chair the meeting or to rotate that role. The meetings follow some basic rules ensuring mutual respect, but are otherwise flexible to accommodate the priorities and wishes of the participants. For the proposed research studies, mental health professionals can be psychiatric community officers, occupational therapists, social workers, nurses, psychiatrists or psychologists.

In Bosnia-Herzegovina, family involvement will be tested in an exploratory RCT where 72 patients with schizophrenia and non-affective psychosis (ICD-10 F20–29) will be recruited and randomly allocated either to family involvement or the control group without family involvement, whilst both groups otherwise continue to receive treatment as usual. In Colombia and Uganda the intervention will be tested in open trials with 30 patients (ICD-10 F20–29, F30-F39) each. In Colombia the trial will be non-controlled. The study design in Uganda includes an additional control site against which patient outcomes will be compared. This control site in Uganda will recruit 30 patients who will continue to receive treatment as usual and will be followed up at the same time points as the intervention participants.

Across all the family involvement studies, the same mental health professional will participate in a maximum of two multi-family groups. This is to avoid any overall effect being dominated by the influence of only one or two individual professionals.

#### Volunteer support

Unpaid volunteers (rather than professionals) are linked with one or more patients to provide psychological, social and practical support, a concept that is often referred to as befriending. The support may be focused on talking, joint activities, expanding social networks or practical support (such as vocational rehabilitation). Volunteer support in befriending programmes is supposed to benefit the patient as well as the volunteer and to strengthen the cohesion and acceptance of patients with mental disorders in the community. Whilst the effects on volunteers themselves and on wider communities have not been systematically studied, there is some evidence suggesting benefits for patients [10–12].

For the proposed research studies, volunteers will be drawn from different backgrounds including university students, the general public and religious communities. All volunteers will be provided with a short training before beginning the intervention and are expected to meet with their matched patients every two weeks for six months.

The study design used to test the volunteer support intervention varies across countries. In Bosnia-Herzegovina, a RCT will be conducted with 72 patients (ICD F20–29) who will be allocated to have one-to-one meetings with a volunteer or to a control group without any volunteer input. Both groups will continue to receive treatment as usual.

Similar to the study on family involvement, the study design for volunteer support in Colombia and Uganda will be open trials with 30 patients. In Colombia the trial will be non-controlled whereas in Uganda patient outcomes will be compared to the aforementioned control group. For both countries, two volunteers will meet small groups of three to six patients. The proposed activities vary: in Uganda the intervention will focus on one or two of the following tasks i) increasing social activity/interactions; ii) engaging in productive, e.g. income-generating, activities; iii) providing practical support for accessing and utilising available professional services and care; and iv) referrals to such services if and when appropriate. It will be up to each group of patients and volunteers to decide which of these tasks to focus on. In Bosnia-Herzegovina and Colombia, the intervention is focused more towards social activities/interactions e.g. visiting parks, museums, and going to cafes. As music and art plays a strong role in the Colombian culture, the social activities may involve producing art and music.

#### Patients and timescale

Patients are eligible if they are 18–65 years of age, have been diagnosed with the type of SMI required in each study, are currently outpatients, and have capacity to provide informed consent. Exclusion criteria are any cognitive impairment that would compromise valid research assessments.

The time period for all interventions will be six months. After the six months intervention period, patients, volunteers, family members and professionals are free to continue with the given intervention, in the original or a different form and in any frequency that the participants may wish to use. After the six months test period the interventions will receive minimal or no support from the research team.

Data collection for all participants in all studies will take place at baseline, at the end of the intervention period (six months) and at follow up (twelve months). All assessments for data collection will be conducted by trained researchers with no involvement in the usual treatment of the patients or the tested interventions. Adherence to the intended intervention will be assessed in all trials.

In the RCTs, clinicians and participants cannot be blinded towards the allocation of the patients to the experimental or control group. However, researchers who conduct the outcome assessments will be blinded to the allocation of patients. Moreover, all baseline assessments will occur prior to randomisation. Randomisation will be conducted by an independent researcher who will use sequential computer generated random numbers to determine allocation. The allocation information will be provided to the unblinded research coordinator at each site. The intervention period begins from the date of randomisation and for all other trials, i.e. non-RCTs, the intervention period begins with the first multi-family meeting or the allocation of patients and volunteers. A summary of the schedule for patients in all DIALOG+ RCTs and Family Involvement and Volunteer Support RCTs in Bosnia-Herzegovina can be found in Tables 2, 3 and 4 respectively.

**Table 2:** Schedule of enrolment, interventions, and assessments for patients in the DIALOG+ study (all sites)

Timepoint	Study period										
	Enrolment	Allocation	Post-allocation								Close out
	*-t*_*1*_	Month 0	*t*_*1 Month 0*_	*t*_*2 Month 1*_	*t*_*3 Month 2*_	*t*_*4 Month 3*_	*t*_*5 Month 4*_	*t*_*6 Month 5*_	*t*_*7 Month 6*_	*Flexible period Month 7–12*	*Month 12*
ENROLMENT:											
Eligibility screen	X										
Informed consent	X										
*MANSA Screening*	X										
Allocation		X									
INTERVENTIONS:											
*DIALOG+*			X	X	X	X	X	X	X	X	
*DIALOG Scale – only in Uganda*			X	X	X	X	X	X	X	X	
*Treatment as Usual – Bosnia and Herzegovina and Colombia*			X	X	X	X	X	X	X	X	
ASSESSMENTS:											
*Socio-demographic Questionnaire*	X										
*MANSA, BPRS, SIX, CSRI, Additional measures (vary by site)*	X								X		X
*Adherence Measures*			X	X	X	X	X	X	X	X

**Table 3:** Schedule of enrolment, interventions, and assessments for patients in the Family Involvement Intervention in Bosnia-Herzegovina

Timepoint	Study period										
	Enrolment	Allocation	Post-allocation								Close out
	*-t*_*1*_	Month 0	*t*_*1 Month 0*_	*t*_*2 Month 1*_	*t*_*3 Month 2*_	*t*_*4 Month 3*_	*t*_*5 Month 4*_	*t*_*6 Month 5*_	*t*_*7 Month 6*_	*Flexible period Month 7–12*	*Month 12*
ENROLMENT:											
Eligibility screen	X										
Informed consent	X										
*MANSA Screening*	X										
Allocation		X									
INTERVENTIONS:											
*Family Involvement Sessions*			X	X	X	X	X	X	X	X	
ASSESSMENTS:											
*Socio-demographic Questionnaire*	X										
*MANSA, BPRS, SIX, CSRI, ITAQ*	X								X		X
*Adherence Measures*			X	X	X	X	X	X	X	X

**Table 4:** Schedule of enrolment, interventions, and assessments for patients of the Volunteer Support in Bosnia-Herzegovina

Timepoint	Study period																
	Enrolment	Allocation	Post-allocation (weeks)														Close out
	*-t*_*1*_	Month 0	*t*_*1 0*_	*t*_*2 2*_	*t*_*3 4*_	*t*_*4 6*_	*t*_*5 8*_	*t*_*6 10*_	*t*_*7 12*_	*t*_*8 14*_	*t*_*9 16*_	*t*_*10 18*_	*t*_*11 20*_	*t*_*12 22*_	*t*_*13 24*_	*Flexible period Month 7–12*	*Month 12*
ENROLMENT:																	
Eligibility screen	X																
Informed consent	X																
*MANSA Screening*	X																
Allocation		X															
INTERVENTIONS:																	
*Volunteer Support Sessions*			X	X	X	X	X	X	X	X	X	X	X	X	X	X	
ASSESSMENTS:																	
*Socio-demographic Questionnaire*	X																
*MANSA, BPRS, SIX, CSRI*	X														X		X
*Adherence Measures*			X	X	X	X	X	X	X	X	X	X	X	X	X	X	

### Measures

Data will be collected using a standardised Case Report Form (CRF) and each partner site will be collecting the same core outcome measures for all recruited patients. All CRFs have been translated into the relevant local language. Country specific CRFs are available upon request.

At baseline, we will obtain *socio-demographic and clinical characteristics* including gender, age, ethnicity, highest level of education, employment, mental and physical diagnoses. Further assessed socio-demographic and clinical data vary slightly across countries to capture the most locally relevant information.

At baseline, six and twelve months we will assess:

*Subjective quality of life* using the Manchester Short Assessment of Quality of Life (MANSA) [13]. The MANSA has been widely used in mental health research and contains satisfaction items with 12 life domains which are rated by the patient between 1 (most negative score) and 7 (most positive score). The mean score of those 12 items is used to reflect subjective quality of life.

In all trials, the MANSA will be used as the primary outcome at six months and in all controlled trials, the MANSA is also used as a screening tool. To avoid including patients with high levels of subjective quality of life at baseline that cannot substantially improve during the study anymore, only patients with a mean score of < 5 are eligible to take part in the study.

*Mental Health Symptoms* will be observer rated on the 24-item Brief Psychiatric Rating Scale (BPRS) [14]. Each symptom is assessed and rated between 1 (not present) and 7 (extremely severe) using a scoring guide. The BPRS guide has been translated into the local languages, and all researchers will be trained in BPRS assessments and interrater-reliability will be established prior to baseline data collection.

*Objective Social Outcomes* will be assessed using the SIX which ranges from 0 (poorest social situation) to 6 (best social situation) and captures whether patients are in employment, have independent accommodation, are living with others and have contacts with friends [15].

*Use of mental health services* will be documented on a simplified version of the Client Service Receipt Inventory (CSRI) [16]. Information on medication use, contact with mental health professionals and any instances of hospitalisation in the last three months will be recorded.

Whilst the above measures are consistently used across countries and studies, further measures are used in only some studies to ensure data collection matches local health priorities. These are listed in Table 5.

**Table 5:** Additional outcome measures to be collected within each country

	Additional Outcome Measures	Bosnia- Herzegovina	Uganda	Colombia
Patients	Internalised Stigma of Mental Illness Inventory [17]	Volunteer Support	All studies	Volunteer Support
	Scale to Assess Therapeutic Relationships in Community Mental Health Care (STAR-P) [18]	DIALOG+		
	Client Satisfaction Questionnaire-8 (at 6 and 12 months) [19]	All studies		
	Self-Esteem Rating Scale [20]	All studies		
	Depression and Anxiety Stress Scale [21]	DIALOG+		
	Insight and Treatment Attitudes Questionnaire (ITAQ) [22]	Family Involvement		
	Medication Adherence Rating Scale [23]		All studies	
Clinicians	Scale to Assess Therapeutic Relationships in Community Mental Health Care (STAR-C) [18]	DIALOG+		
Family Members/Friends	Burden Scale for Family Caregivers [24]	Family Involvement		
	Community Attitudes Towards Mental Illness Scale [25]	Family Involvement		
	Zarit Burden Interview [26]		Family Involvement	
Volunteers	Community Attitudes Towards Mental Illness Scale [25]	Volunteer Support	Volunteer Support	
	Social Distance Questionnaire [27]	Volunteer Support		
	Self-esteem Rating Scale [20]	Volunteer Support		

### Sample size calculations

Formal sample size calculations were conducted for the DIALOG+ RCTs in Uganda and Colombia in order to detect a medium effect size of 0.5, setting power at 80% for 5% significance. The total number of patients required is 64 per group (*n* = 128). After accounting for clustering of patients treated by the same clinician, based on an Intra-Cluster-Coefficient of 0.01 as observed within the DIALOG+ trial in the UK [28], a conservative design effect of 1.04, and allowing for a drop-out rate of 17%, a total of 168 patients will need to be recruited to give an analysable sample of 140 (70 per group).

Estimates for exploratory randomised controlled trials vary, but it has been suggested that a minimum of 30 participants per arm are required [29]. Therefore in Bosnia-Herzegovina, the sample size for all RCTs include 36 per arm, to allow for a 20% drop out rate which has been demonstrated in previous research with individuals with psychosis [30].

For all other controlled and non-controlled trials, we plan a sample size of 30 participants per arm or in total respectively. Central limit theory suggests 30 as a minimum sample size to enable meaningful parameter estimates [31, 32].

The plan to have larger studies on DIALOG+ than on family involvement and volunteer support reflects a particular interest of stakeholders in each country who felt that DIALOG+ may play an important role in the further development and establishment of community mental health care in the participating countries.

#### Experiences with the interventions

At the end of the six month intervention period, a subset of participants (patients, clinicians, family members/friends and volunteers) who were allocated to the interventions will complete semi-structured, in-depth interviews about their experiences of receiving the interventions, with unblinded researchers. Purposive sampling will be employed to capture patients with varying levels of engagement with their clinician/volunteer/family group and with different outcomes. The interviews will be audio recorded, transcribed and analysed using the thematic analysis framework proposed by Braun and Clarke [33].

#### Commonalities and differences

Whilst the type of interventions, target groups of patients with SMI, timelines and core outcome criteria are consistent across all studies in all countries, there are differences in the precise diagnostic inclusion criteria, the study designs and some outcome criteria. The most important commonalities and differences of the studies in the three countries are summarised in Table 6

**Table 6:** Similarities and differences across the nine research studies in three LMICs

Consistent	Variable
Intervention period for 6 months	
Principles of interventions	Details of interventions
Patients with severe mental illness	Precise diagnostic groups
Outcome assessments	Trial designs:
- Baseline - 6 months (post-intervention) - 12 months	- Non-controlled, non-randomised controlled and RCTs - Sample sizes - Blinded and non-blinded
Core outcome criteria:	Further outcome criteria includes:
- Objective social situation (SIX)	- Caregiver burden
- Subjective quality of life (MANSA)	- Stigma
- Symptoms (BPRS)	- Medication adherence
- Service use (CSRI adapted)	- Self esteem

#### Settings

In Bosnia and Herzegovina, all research activities will take place at the Clinical Centre University of Sarajevo.

In Colombia, the DIALOG+ study will take place at various sites in Bogotá (San Ignacio hospital, Clínica la Inmaculada, Clínica Fray Bartolomé) and Hospital Departamental Psiquiátrico Universitario del Valle ESE, Cali. The Family involvement and Volunteer support studies will recruit patients mainly from the Association of Persons with Schizophrenia and their Families and the Bipolar Patients Association, but they will also accept referrals from the participating research sites in Bogotá.

In Uganda, the DIALOG+ study will take place in Kampala at Butabika Mental Health Hospital and its surrounding outreach clinics. Three mental health clinics (or centres) in different areas of the country were randomised to one of three arms: Family involvement study (Masaka), Volunteer support study (Jinja) and Control (Mitiyana).

#### Data management and analysis

All data will be collected on paper-based CRFs and pseudonymised data will be entered onto a secure shared electronic database, REDCap, accessible to all partners. The electronic database will contain validations to ensure accurate data entry. Local coordinators and the UK team will provide regular monitoring to ensure accurate data collection and entry. All patient-identifiable data will be pseudonymised and password-protected at the local research site.

The studies will first be analysed separately, where the mean MANSA score will be compared before and after the intervention as well as between intervention and control arms (for all controlled studies). We will also combine the datasets. The consistencies in the interventions and study designs will make it possible to compare findings and experiences and learn from commonalities and differences. Detailed statistical analysis plans will be completed and signed off by all relevant partners prior to any data analysis or unblinding of the data. Once signed off, the plans will be available on request. The director of the Group will have access to the final dataset.

#### Monitoring

An overall Advisory Group has been established for the whole research programme of the NIHR Global Health Research Group. It comprises members who are independent of the organisations involved in the research. Members have expertise in mental health care in the partner countries, global mental health, research methodology and implementation of study designs, including RCTs, in community settings. Their role is equivalent to a Steering Group and covers the research programme as a whole, as well as individual studies. A formal Data Monitoring Committee was not established as this function will be covered by the Advisory Group, who will provide input on data monitoring, if and as required.

#### Dissemination

The findings will be disseminated to national stakeholders, including policy makers, mental health workers and the general public. Dissemination events will be held in each country. The findings will be shared with the scientific community in national and international conferences and in open access publications. The general availability of the data sets varies in line with the conditions set by local ethics committees and research governance rules. Yet, all data sets will be made available upon request subject to data sharing agreements.

In addition to the findings of the studies themselves, we will also publish reflective articles about the experiences with refining interventions and study designs in partnership with national stakeholders and conducting studies with similarities and differences across three LMICs.

## Discussion

The approach of the research group to establish a partnership across countries on three continents and explore resource-oriented interventions for community mental health care for people with SMI has led to nine separate study protocols. The content and design of the studies were developed in workshops, consultations and discussion groups at all sites. In each LMIC, each considered intervention was discussed in depth and in partnership with local stakeholders considering the local context, healthcare priorities, practicalities and logistics of conducting the research studies, as well as potential study sites, participants and study designs. Workshop attendees included different groups of mental health professionals, researchers, government officials and representatives, volunteers, peer support workers, representatives from patient and family organisations, non-government organisations and student organisations. These events and regular on-going communication among all partners led to protocols for the three types of resource-oriented interventions and for studies testing them.

The study designs which are non-controlled or non-randomised may provide limited conclusions on effectiveness whilst the exploratory RCTs have relatively small sample sizes providing limited statistical power for detecting effects. However, we believe they will provide valuable insight into the feasibility and acceptability of such interventions in the relevant contexts.

The protocols show substantial consistencies across the three LMICs but at the same time, there are a number of variations reflecting different contexts of national health and social care systems and the specific priorities and wishes of national stakeholders. This approach is distinct from a conventional multi-centre study and required excellent collaboration and communication both across and within the partner countries. Each of the nine studies will primarily be analysed separately. Yet, the consistencies in the interventions and the study designs will make it possible to compare findings and learn from commonalities and differences in the findings and experiences. To our knowledge, this is an unusual and somewhat innovative approach in global mental health research. The results of the studies may show to what extent such designs across several countries do or do not reveal additional insights. The approach also ensured a relevance to the different national contexts so that the findings can potentially impact on health policies and clinical practice in each country.

Through these research studies and activities, the Group aims to encourage sharing and learning across contexts thereby building sustainable research capacity and exploring and deepening the understanding of global mental health.

## Abbreviations

BPRS: Brief Psychiatric Rating Scale
CRF: Case Report Form
CSRI: Client Service Receipt Inventory
HICs: High-income countries
ICD-10: International Classification of Diseases, Tenth Revision, Clinical Modification
ISRCTN: International Standard Randomised Controlled Trials Number
ITAQ: Insight and Treatment Attitudes Questionnaire
LMICs: Low- and middle-income countries
MANSA: Manchester Short Assessment of Quality of Life
MNS: Mental, Neurological and Substance misuse disorders
NIHR: National Institute of Health Research
ODA: Official Development Assistance
RCT: Randomised controlled trial
SIX: Objective Social Outcomes Index
SMI: Severe mental illness
TAU: Treatment as usual
UK: United Kingdom

## References

[1] Patel V, Prince M (2010). Global mental health: a new Global Health field comes of age. JAMA..

[2] Patel V, Thornicroft G (2009). Packages of care for mental, neurological, and substance use disorders in low- and middle-income countries: PLoS medicine series. PLoS Med.

[3] Priebe S, Omer S, Giacco D, Slade M (2014). Resource-oriented therapeutic models in psychiatry: conceptual review. Br J Psychiatry.

[4] Priebe S, Kelley L, Omer S, Golden E, Walsh S, Khanom H (2015). The effectiveness of a patient-centred assessment with a solution-focused approach (DIALOG+) for patients with psychosis: a pragmatic cluster-randomised controlled trial in community care. Psychother Psychosom.

[5] Priebe S, Golden E, Kingdon D, Omer S, Walsh S, Katevas K, et al. Effective patient-clinician interaction to improve treatment outcomes for patients with psychosis: a mixed methods design (DIALOG+). NIHR Journals Library, Heal Technol Assess Program Grants Appl Res. 2017;5(6). 28252893

[6] Omer S, Golden E, Priebe S (2016). Exploring the mechanisms of a patient-centred assessment with a solution focused approach (DIALOG+) in the community treatment of patients with psychosis: a process evaluation within a cluster-randomised controlled trial. PLoS One.

[7] The World Health Organization. The ICD-10 classification of mental and Behavioural disorders. Geneva; 1993.

[8] Kaselionyte J, Dirik A, Tulloch S, Priebe S, Giacco D (2016). Psychosis seminars: an open forum for service users, carers and professionals. BJPsych Open.

[9] MacGabhann L, Dunne S, McGowan P, Amering M (2018). Democratic communities: evaluating trialogue for mental health stakeholders. Ment Heal Rev J.

[10] Siette J, Cassidy M, Priebe S (2017). Effectiveness of befriending interventions: a systematic review and meta-analysis. BMJ Open.

[11] Toner S, Cassidy M, Chevalier A, Farreny A, Leverton M, Pinto da Costa M, et al. Preferences for befriending schemes: a survey of patients with severe mental illness. BMC Psychiatry. 2018;18(64). 10.1186/s12888-018-1643-9 PMC5845380 29523114

[12] Thompson R, Valenti E, Siette J, Priebe S (2015). To befriend or to be a friend: a systematic review of the meaning and practice of “befriending” in mental health care. J Ment Health.

[13] Priebe S, Huxley P, Knight S, Evans S (1999). Application and results of the Manchester short assessment of quality of life (MANSA). Int J Soc Psychiatry.

[14] Ventura J, Lukoff D, Nuechterlejn KH, Liberman RP, Green MF, Shanea A (1993). Brief psychiatric rating scale (BPRS) expanded version (4.0). Scales.

[15] Priebe S, Watzke S, Hansson L, Burns T (2008). Objective social outcomes index (SIX): a method to summarise objective indicators of social outcomes in mental health care. ActaPsychiatrScand..

[16] Beecham J, Knapp M, Thornicroft G (2001). Costing psychiatric interventions in measuring mental health needs. 2nd ed.

[17] Hammer Joseph H., Toland Michael D. (2017). Internal structure and reliability of the Internalized Stigma of Mental Illness Scale (ISMI-29) and Brief Versions (ISMI-10, ISMI-9) among Americans with depression. Stigma and Health.

[18] McGuire-Snieckus R, McCabe R, Catty J (2007). A new scale to assess the therapeutic relationship in community mental health care: STAR. Psychol Med.

[19] Nguyen T, Attkisson C, Stegner B (1983). Assessment of patient satisfaction: development and refinement of a service evaluation questionnaire. Eval Progr Plann.

[20] Lecomte T, Corbiere M, Lasine F (2006). Investigating self-esteem in individuals with schizophrenia: relevance of the self-esteem rating scale-short form. Psychiatry Res.

[21] Lovibond SH, Lovibond PF (1995). Manual for the depression anxiety stress scales. 2nd ed.

[22] Mohamed S, Rosenheck R, McEvoy J, Swartz M, Stroup S, Lieberman J (2008). Cross-sectional and longitudinal relationships between insight and attitudes toward medication and clinical outcomes in chronic schizophrenia. Schizophr Bull.

[23] Thompson K, Kulkarni J, Sergejew A (2000). Reliability and validity of a new medication Adherance rating scale (MARS) for the psychoses. Schizophr Res.

[24] Gräsel E, Chiu T, Oliver R. Development and validation of the Burden Scale for Family Caregivers (BSFC) Comprehensive Rehabilitation and Mental Health Services, [Internet]. Toronto, Ontario, Canada; 2003. Available from: http://www.psychiatrie.uk-erlangen.de/fileadmin/einrichtungen/psychiatrie/bilder/graesel/Gr%E4sel_Chiu_etal_2003.pdf

[25] Taylor S, Dear M (1981). Scaling community attitudes toward the mentally ill. Schizophr Bull.

[26] Hérbert R, Bravo G, Préville M (2000). Reliability, validity, and reference values of the Zarit burden interview for assessing informal caregivers of community-dwelling older persons with dementia. Can J Aging.

[27] Schomerus G, Van der Auwera S, Matschinger H (2015). Do attitudes towards persons with mental illness worsen during the course of life? An age-period-cohort analysis. Acta Psychiatr Scand.

[28] Priebe S, Kelley L, Golden E, McCrone P, Kingdon D, Rutterford C (2013). Effectiveness of structured patient-clinician communication with a solution focused approach (DIALOG+) in community treatment of patients with psychosis - a cluster randomised controlled trial. BMC Psychiatry..

[29] Browne R (1995). On the use of a pilot sample for sample size determination. Stat Med.

[30] Szymczynska P, Walsh S, Greenberg L, Priebe S (2017). Attrition in trials evaluating complex interventions for schizophrenia: systematic review and meta-analysis. J Psychiatr Res.

[31] Le Cam L (1986). The central limit theorem around 1935. Stat Sci.

[32] Davidson J (1994). The central limit theorem, stochastic limit theory.

[33] Braun V, Clarke V (2006). Using thematic analysis in psychology. Qual Res Psychol.

